# Genetic mapping identifies loci that influence tomato resistance against Colorado potato beetles

**DOI:** 10.1038/s41598-018-24998-5

**Published:** 2018-05-09

**Authors:** Erandi Vargas-Ortiz, Itay Gonda, John R. Smeda, Martha A. Mutschler, James J. Giovannoni, Georg Jander

**Affiliations:** 1 Boyce Thompson Institute, Ithaca, New York 14853 USA; 2 Department of Plant Breeding and Genetics, Cornell University, Ithaca, NY 14853 USA; 3 USDA Robert W. Holley Center for Agriculture and Health, Ithaca, New York 14853 USA; 4 Present Address: Department of Molecular Biology, Institute for Scientific and Technological Research of San Luis Potosí, San Luis Potosí, Mexico

## Abstract

The Colorado potato beetle (CPB; *Leptinotarsa decemlineata* Say), the most economically important insect pest on potato (*Solanum tuberosum* L.), also feeds on other Solanaceae, including cultivated tomato (*Solanum lycopersicum* L.). We used tomato genetic mapping populations to investigate natural variation in CPB resistance. CPB bioassays with 74 tomato lines carrying introgressions of *Solanum pennellii* in *S. lycopersicum* cv. M82 identified introgressions from *S. pennellii* on chromosomes 1 and 6 conferring CPB susceptibility, whereas introgressions on chromosomes 1, 8 and 10 conferred higher resistance. Mapping of CPB resistance using 113 recombinant inbred lines derived from a cross between *S. lycopersicum* cv UC-204B and *Solanum galapagense* identified significant quantitative trait loci on chromosomes 6 and 8. In each case, the *S. galapagense* alleles were associated with lower leaf damage and reduced larval growth. Results of both genetic mapping approaches converged on the same region of chromosome 6, which may have important functions in tomato defense against CPB herbivory. Although genetic mapping identified quantitative trait loci encompassing known genes for tomato acyl sugar and glycoalkaloid biosynthesis, experiments with acyl sugar near-isogenic lines and transgenic *GAME9* glycoalkaloid-deficient and overproducing lines showed no significant effect of these otherwise insect-defensive metabolites on CPB performance.

## Introduction

The Colorado potato beetle (CPB, *Leptinotarsa decemlineata* Say) has become the most important insect pest on cultivated potato (*Solanum tuberosum* L.). Both adult beetles and larvae feed on potato leaves. A CPB female can lay around 3,000 eggs in a three-month lifespan^1^, and larvae feed for about 20 days on their host plants before pupation. Thus, in untreated fields, CPB can completely destroy potato crops^2^. Moreover, CPB readily develops insecticide resistance^3,4^ and resistant CPB populations are spreading rapidly through potato-growing regions of the United States^5^. Although CPB is mainly a pest on *S. tuberosum*, it also can feed and complete its life cycle on other plants in the Solanaceae family, including eggplant (*Solanum melongena* L.) and tomato (*Solanum lycopersicum* L.)^6^. CPB has been shown to reduce tomato production in the field^7^, with young plants being the most severely affected^8,9^. Furthermore, CPB has the potential to become better-adapted to tomato and thus become a more severe tomato pest^10^.

Tomato introgression lines developed by Eshed and Zamir^11^ have a series of introgressions of the *Solanum pennellii* Correll (LA0716) genome in the background of *S. lycopersicum* cv. M82. As these lines have introgressions of defined segments of the *S. pennellii* genome into *S. lycopersicum*, phenotypic variation in the introgression lines relative to M82 can be associated with a specific chromosomal segment^11^. The *S. pennellii* introgression population, which was developed to study the genetic components of tomato yield and fruit quality, has been widely used for mapping other important quantitative trait loci (QTL) related to biotic and abiotic stress responses. By 2013, at least 3069 QTLs had been identified using the *S. pennellii* introgression lines^12^, including ones for fruit biochemistry, yield, fitness, salt tolerance, and antioxidant capacity^13,14^. Tomato defensive traits that have been mapped with these introgression lines include the diversity of compounds synthetized by trichomes^15^, production of monoterpenes^16^, biosynthetic enzymes for acylsugars^17–19^, and the glycoalkaloid biosynthetic pathway^20,21^.

Tomato recombinant inbred lines (RILs), which are generated by repeated selfing of F2 progeny derived from a cross between two tomato species also have been used to identify QTLs. RILs derived from a cross of *S. lycopersicum* with *Solanum galapagense* S.C. Darwin & Peralta were used to identify QTLs related to fruit quality (fruit weight and soluble sugars) and morphological traits^22,23^. RILs originating from a cross of *S. lycopersicum* × *Solanum cheesmaniae* (L.Riley) Fosberg, which is very close genetically to *S. galapagense*^24^, identified QTLs related to salt stress tolerance^25^ and rootstock effects that help to elucidate the physiological mechanisms behind salt tolerance^26,27^. Experiments with RILs derived from *S. lycopersicum* × *Solanum pimpinellifolium* L. identified acylsugar content and trichomes as resistance traits against two-spotted spider mites (*Tetranychus urticae* C.L. Koch)^28^.

The ability of CPB to grow on tomato plants can be used to study natural variation in resistance against this pest, with the ultimate goal of increasing pest resistance in the field. In particular, genetic mapping populations facilitate the identification of tomato loci that provide enhanced resistance to CPB feeding. In this study, we analyzed tomato resistance variation against CPB in *S. lycopersicum* cv. M82 × *S. pennellii* LA0716 introgression lines, as well as in RILs derived from *S. lycopersicum* cultivar UC-204B × *S. galapagense* LA0483^22,29^, with the objective being the genetic mapping of QTLs that provide CPB resistance.

## Results

### Colorado potato beetle performance on *S. pennellii* and *S. lycopersicum* parental lines

**Figure 1:**
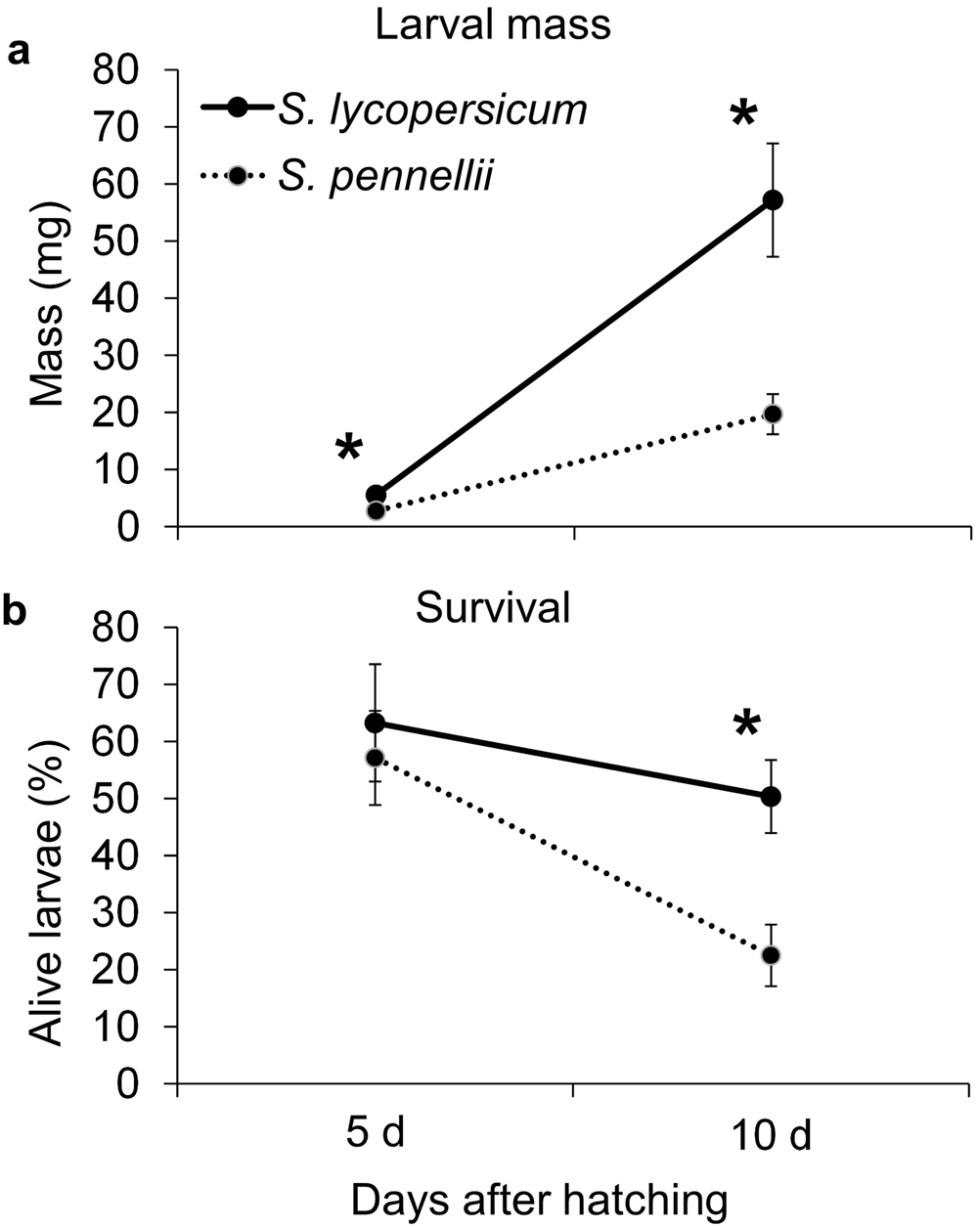
Colorado potato beetle performance on parental tomato plants. (**a**) CPB larval mass and (**b**) survival on *S. lycopersicum* (solid line) or *S. pennellii* (dashed line). Values are means ± SE of 7 and 9 plants, respectively. *P < 0.05 by Student’s t-test.

CPB larval mass was lower on *S. pennellii* plants than on *S. lycopersicum* M82 plants at both 5 and 10 days after the start of infestation (Fig. 1a). Differences in survivorship were found only after ten days of feeding, when CPB survival was lower on *S. pennellii* (Fig. 1b). Among the surviving insects, adult mass (*p = *0.08, *t-*test) and adult emergence rate (p = 0.06, *t-*test) also tended to be lower in *S. pennellii* plants, but these effects were not significant at the p < 0.05 level.

### Colorado potato beetle feeding on *S. pennellii* introgression lines

**Figure 2:**
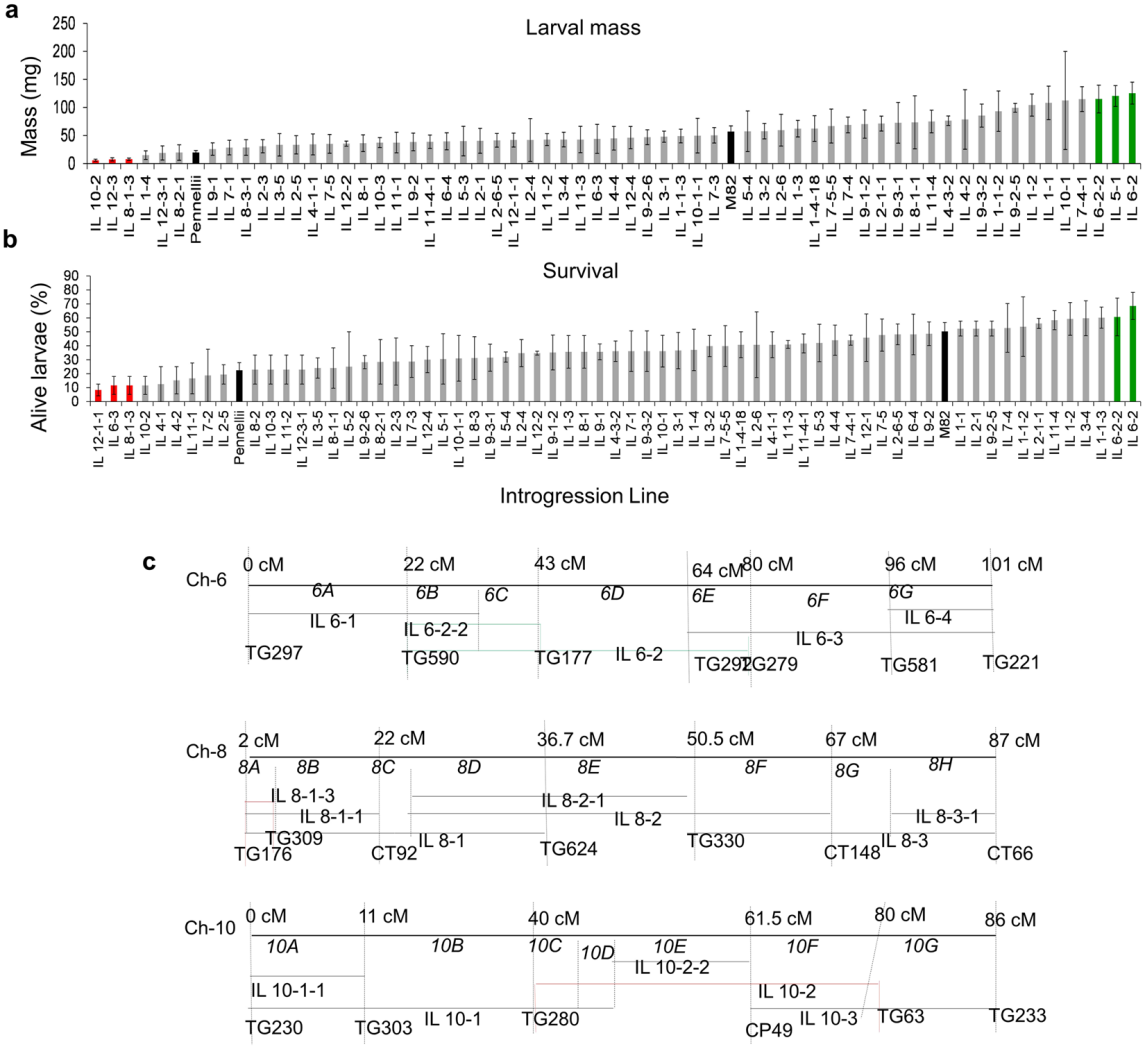
Colorado potato beetle performance on *S. pennellii* introgression lines. (**a**) CPB larval mass and (**b**) survivorship were recorded 10 days after infestation. Black bars show performance and survival on parental lines. Green bars represent introgression lines where CPB has either significantly better performance than on M82 or greater survival than on *S. pennellii* (P** < **0.05, Dunnett**’**s test). Red bars represent introgression lines where CPB has the same performance or survivorship as on *S. pennellii* plants and lower than on M82 (P < 0.05, Dunnett**’**s test). All bars represent the mean of 2 or 3 plants. (**c**) Representation of the chromosomes with introgression lines that affect CPB resistance. Each chromosome is divided into bins based on the classic tomato genetic map (marked with italics, *6 A, 6B*, etc.) Horizontal bars are marked with the name of the respective introgression lines carrying segments of *S. pennellii* introgressed into *S. lycopersicum*. The approximate position of the flanking markers is shown in centimorgans (Tomato Expen 2000 map, www.solgenomics.net). The most susceptible (IL 6-2, IL 6-2-2, green) and the most resistant (IL 8-1-3, IL 10-2; red) lines are indicated by colored bars.

As differences in CPB performance on *S. pennellii* introgression lines were greater after 10 days of plant growth than on younger seedlings, this growth stage was used to investigate the genetic basis of tomato defense against CPB. After 10 days of feeding, larval mass ranged from 5 mg to 142 mg (Fig. 2a), whereas survival ranged from 8% to 68% in the different *S. pennellii* introgression lines (Fig. 2b). A positive correlation between larval mass and survival (Kendall’s correlation rank, τ = 0.3, *p* < 0.01) was found. Lines where CPB had significantly lower larval mass or survival compared to M82 (Fig. 2, red bars), as well as lines where larval mass or survival was greater than on M82 in lines (Fig. 2, green bars) were identified. Introgressions causing both significantly lower larval mass and survival were found on chromosomes 8 (IL 8-1-3) and 10 (IL 10-2). Susceptible *S. pennellii* introgressions causing both greater larval mass and survival were found on chromosome 6 (overlapping introgressions IL 6-2 and IL 6-2-2).

### Colorado potato beetle feeding on *S. lycopersicum* × *S. galapagense* recombinant inbred lines

**Figure 3:**
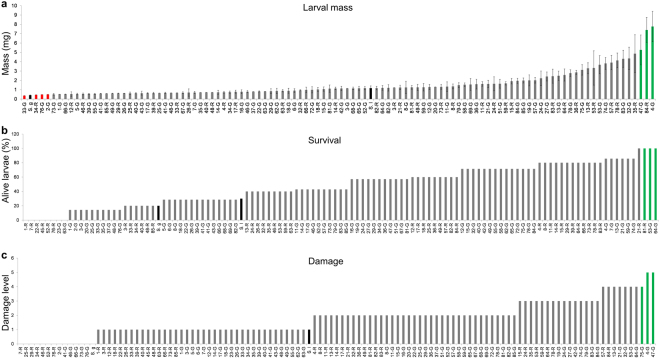
Variation in Colorado potato beetle performance on *Solanum lycopersicum* × *S. galapagense* recombinant inbred lines. (**a**) Average mass of surviving CPB larvae on each leaflet, ±SE if at least two larvae survived; (**b**) Percent survival of seven larvae that were placed on each leaflet. (**c**) Damage in the leaflet was ranked from 0 (no damage) to 5 (50% damage) and the number of RILs per damage level recorded after five days of feeding. Black bars show performance and survival on the parental lines (*S. lycopersicum*, *S.l*; *S. galapagense*, *S.g*). Green bars represent individuals where CPB performance and survival were the highest, red bars represent recombinant inbred lines where CPB performance and survival were the lowest. Each bar represents an assay conducted with an individual leaflet.

After 5 days of feeding on *S. lycopersicum* × *S. galapagense* RIL leaflets, CPB larval mass ranged from 0.4 to 3.7 mg (Fig. 3a), whereas survival ranged from 0 to 100% (Fig. 3b). There was a positive correlation between larval weight and survival (Kendall´s correlation rank, τ = 0.3, *p* < 0.001). Damage in the leaflets varied as show in Fig. 3c and was positively correlated with larval weight (τ = 0.6, *p* < 0.001) and survival (τ = 0.3, *p* < 0.001). There is evidence for transgressive segregation in the population, as CPB larvae performed significantly better on the most sensitive RILs than on either of the parents.

**Figure 4:**
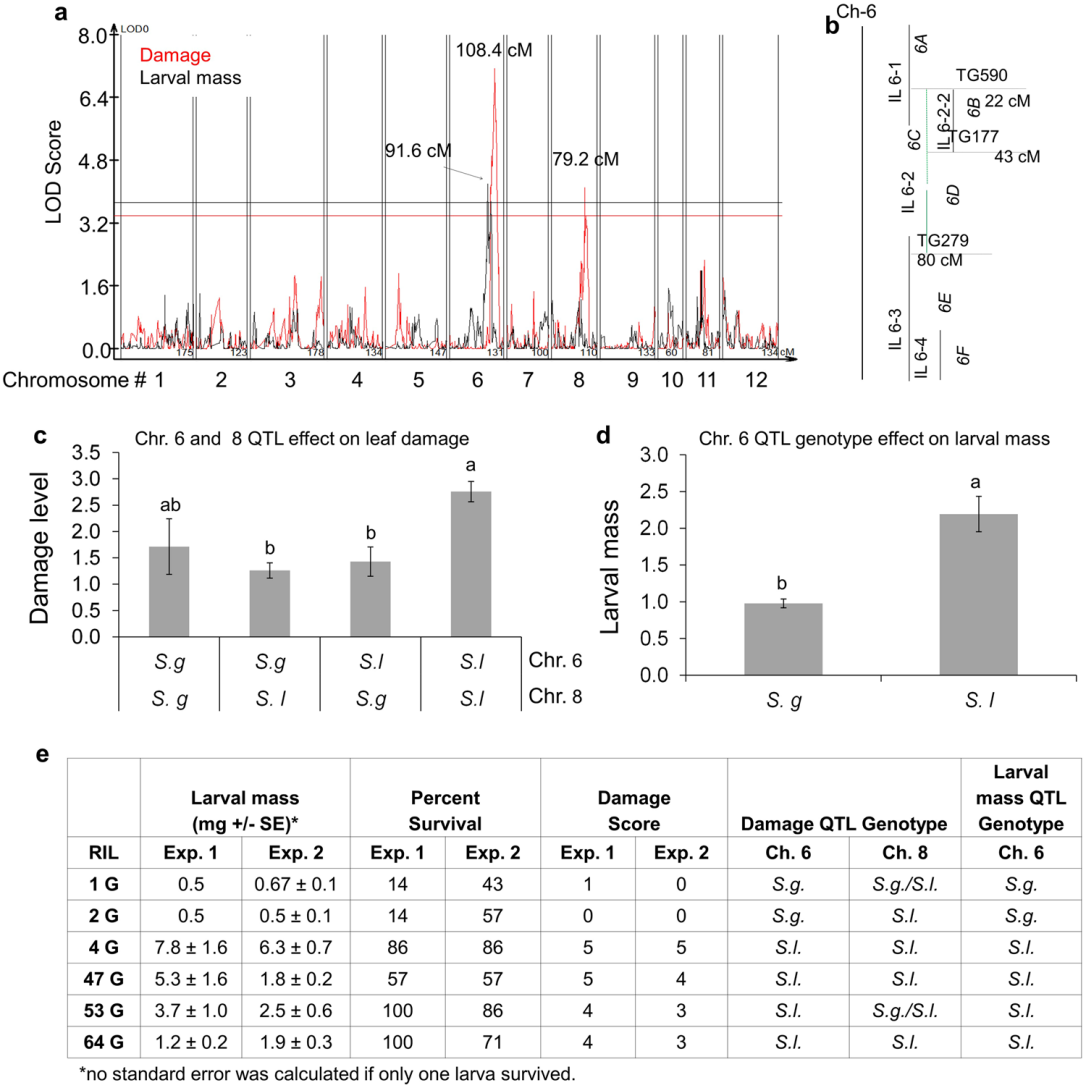
Quantitative trait loci for *S. galapagense* RILs. (**a**) Two QTLs for leaf damage (red line) were found, one on chromosome 6 and one on chromosome 8. A larval mass QTL (black line) was found on chromosome 6. A 95% confidence level was used. (**b**) Representation of the common region between susceptible IL 6-2 and the QTLs on chromosome 6. The dashed line contains the larvae mass QTL on BIN *6D*, and part of the leaf damage QTL in BIN *6E*. The approximate position of the flanking markers is shown in centimorgans of the *S. lycopersicum* genetic map (Tomato Expen 2000 map, www.solgenomics.net). (**c**) Leaf damage in RILs that were grouped on the basis of having the *S. lycopersicum* (*S.l*.) or *S. galapagense* (*S.g*.) alleles for the QTL on chromosomes 6 or 8. Bars represent means ± SE. Different letters indicate significant differences, p < 0.05, HSD test. (**d**) Larval mass in RILs separated by having the *S. lycopersicum* (*S.l*.) or *S. galapagense* (*S.g*.) allele for the QTL on chromosomes 6. Different letters indicate significant differences, p < 0.05, HSD test. (**e**) Larval mass, percent survival, and damage score from an independent confirmation of recombinant lines that showed high (1 G and 2 G) or low (4 G, 47 G, 53 G, and 64 G) CPB resistance in Fig. 3. The plant genotype, *S. lycopersicum* (*S.l*.), *S. galapagense* (*S.g*.), or heterozygous (*S.g./S.l*.) at the site of the identified resistance QTL is indicated.

Single nucleotide polymorphism (SNP) marker data for the *S. lycopersicum* × *S. galapagense* RIL population were obtained from RNAseq data (Supplementary Table 1, positions refer to tomato reference genome build 2.50). Composite interval mapping showed two QTLs with non-additive effects for leaf damage level (Fig. 4a, red line) on chromosomes 6 and 8 that explain 21% and 11% of the total variance for this trait, respectively. The chromosome 6 QTL region overlaps with *S. pennellii* IL 6-2 (Fig. 4b), which also affects CPB resistance. RILs with at least one S. *galapagense* (*S.g*.) allele had approximately 0.5 units less damage than RILs with both *S. lycopersicum* (*S.l*.) alleles (Fig. 4c). A QTL explaining 13% of the variance in larval mass (Fig. 4a, black line) was also found in chromosome 6. On average, larvae that fed on RILs with the *S. lycopersicum* (*S.l*.) allele had double the mass of ones that fed on RILs with the *S. galapagense* (*S.g*.) allele (Fig. 4d). Independent experiments were conducted with the most CPB-resistant RILs (1-G and 2-G) and the most CPB-sensitive RILs (4-G, 47-G, 53-G, and 64-G) to show that larval mass, survival, and leaf damage (Fig. 4e) are consistent with the original RIL assays (Fig. 3). These results are also consistent with the predicted effects of the RIL genotypes for the QTL on chromosomes 6 and 8.

The leaf damage QTL on chromosome 6 is in a 1.73 Mb region with 237 annotated genes (Supplementary Table 2). On chromosome 8, the leaf damage QTL is located in a 640 kb region with 76 annotated genes (Supplementary Table 3) This location on chromosome 8 is distinct from the resistance locus identified with *S. pennellii* introgression lines (IL 8-1-3; Fig. 2c). The larval mass QTL is in a 1.12 Mb region with 150 annotated genes (Supplementary Table 4), which overlaps with *S. pennellii* IL 6-2 BIN 6-D. No significant QTL was found for CPB survival.

Analysis of the known genes in the three QTL mapping regions shows that there are several with potential roles in plant defense. These include MYB transcription factors, Kunitz trypsin inhibitors, and genes involved in ethylene responses. Other genes in the three genetic mapping intervals were differentially expressed in prior experiments to investigate tomato defense responses^30–34^, including genes that had altered expression after *T. urticae* infestation^30^, flagellin treatment^31^, or effector triggered immunity^32^.

### Effect of acylsugars on Colorado potato beetles

**Figure 5:**
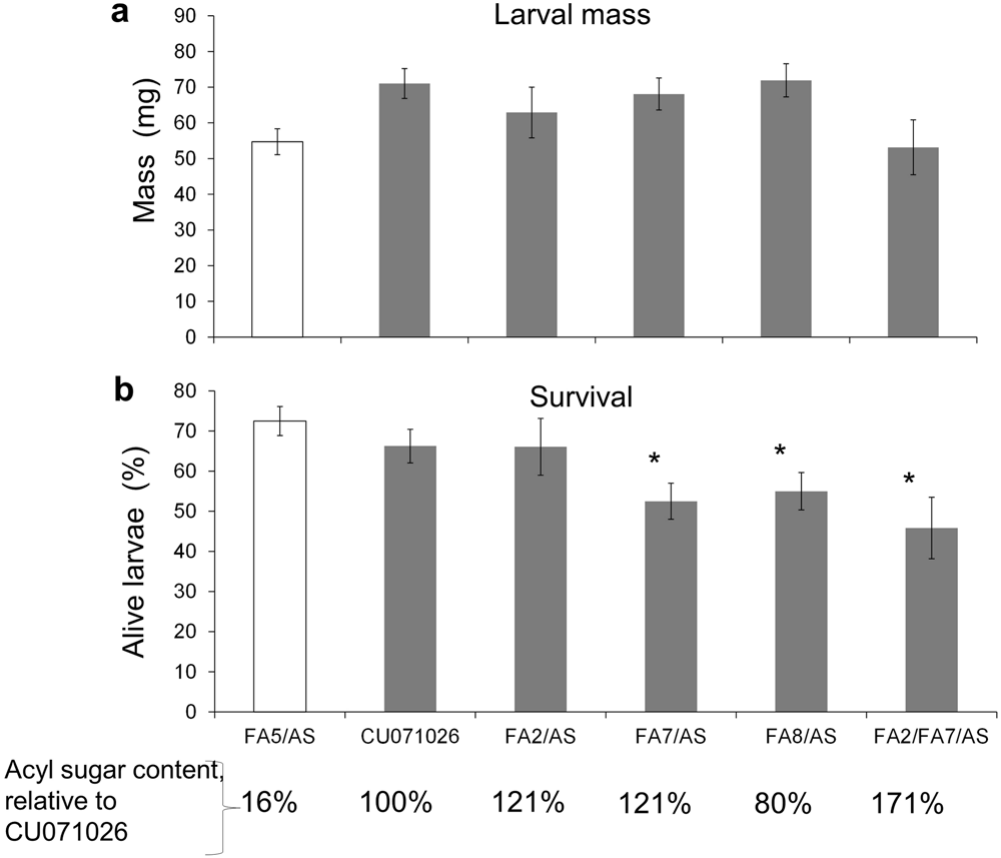
Colorado potato beetle performance on acyl sugar lines after 10 days of feeding. (**a**) Larval mass and (**b**) survival were recorded after 10 days of feeding. Bars represent the mean of ten plants ± SE. * p < 0.05, Dunnett’s test using FA5/AS as a low/negative-acylsugar control.

Acylglucoses of *S. pennellii* are both deterrent and had a negative effect on survival and development of two generalist lepidopteran herbivores, corn earworm (*Helicoverpa zea* Boddie) and beet armyworm (*Spodoptera exigua* Hübner)^35^. To determine whether acylsugars also function in tomato defense against CPB, larval mass and survival, adult emergence rate, and adult mass, were assessed on five backcrossed introgression lines with variable acylsugar content^36,37^. CPB larval mass was not significantly affected by ten days of feeding in the acylsugar lines (F = 0.69 *p* = 0.6) (Fig. 5a). However, there was an effect on larval survival (F = 3.7, *p* = 0.006). Larval survival was lower in FA7/AS (27% lower), FA8/AS (24% lower) and FA2/FA7/AS (36% lower) lines compared to line FA5/AS (Fig. 5b). At the adult stage, there was no effect of acylsugars on adult mass (F = 0.69, *p* = 0.6). Adult emergence rate was affected by the genotype (F = 2.1, *p* = 0.03), but adult emergence was not significantly different on any of the acylsugar lines when compared to that of FA5/AS (Table 1).

**Table 1:** Dunnett’s test for adult emergence on acylsugar lines.

Comparison	Estimate	Std. Error	t value	Pr (>|t|)
CU071026 - FA5/AS = = 0	7.5	7.2	1.0	0.76
FA2/AS - FA5/AS = = 0	−2.14	7.93	0.27	0.99
FA7/AS - FA5/AS = = 0	−16.25	7.20	−2.26	0.11
FA8/AS - FA5/AS = = 0	−7.5	7.20	−1.04	0.76
FA2/7/AS - FA5/AS = = 0	−9.17	8.31	−1.10	0.71

### Effect of α-tomatine on Colorado potato beetles

**Figure 6:**
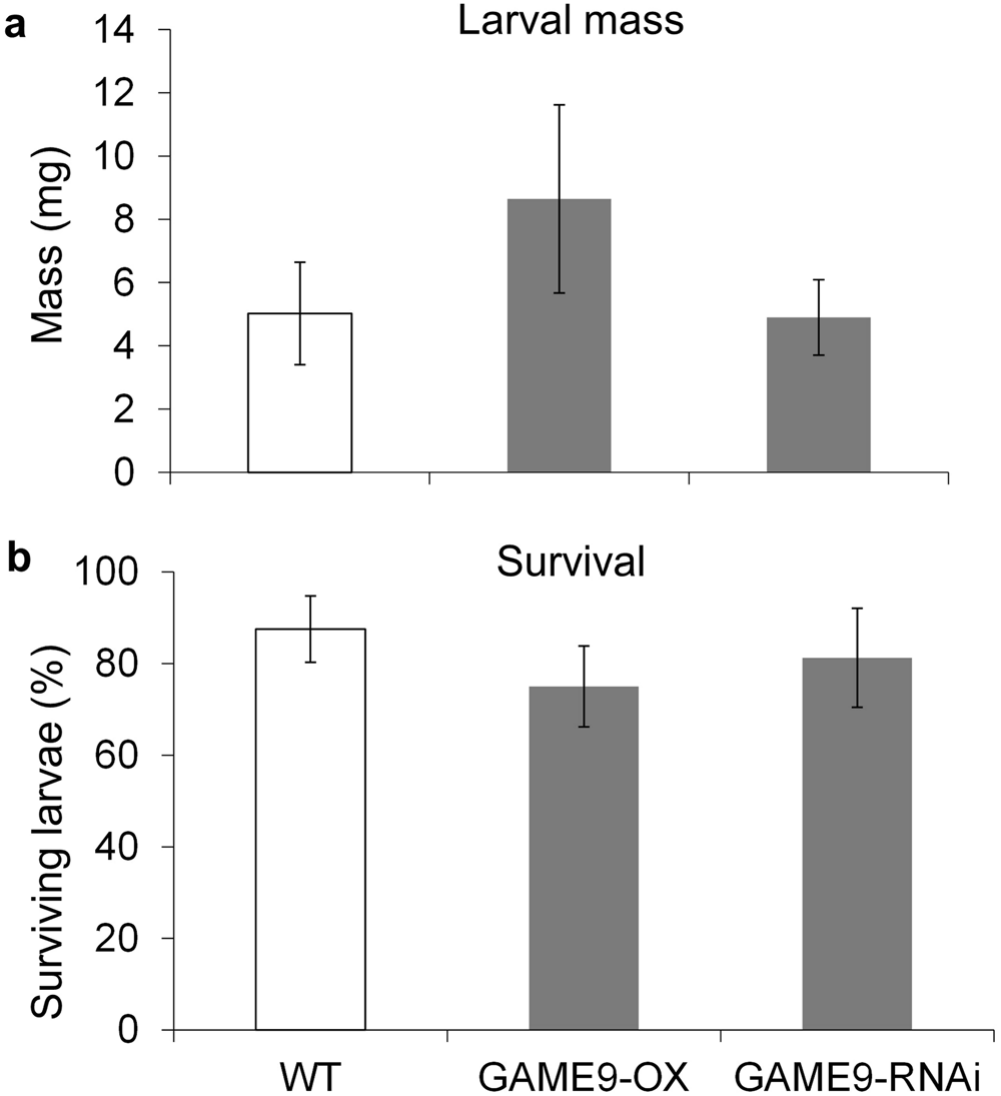
Effect of tomatine content on CPB performance. (**a**) Colorado potato beetle larval mass and (**b**) survival were recorded after 5 days of feeding on *S. lycopersicum* var. Micro Tom (WT, n = 4), and *GAME9* overexpressing (*GAME9*-OX, n = 5) and silenced (*GAME9*-RNAi, n = 4) plants. Bars are mean ± SE. There are no significant differences; P > 0.05, ANOVA.

Recently Cardenas *et al*. showed that *GAME9*, which encodes an AP2/ERF-type transcription factor regulating the biosynthesis of glycoalkaloids in tomato and potato, is located on chromosome 1 between TG21 and TG59 markers and forms part of a cluster of ERF-genes^20^. The location of this cluster overlaps with *S. pennellii* IL 1-1(accession 4028) and IL 1-2 (accession 4021) which were susceptible to CPB in our bioassays (Fig. 2).To determine whether tomatine has a detrimental effect of on CPB larvae, experiments were conducted using tomato plants (*S. lycopersicum* var. Micro Tom) with the *GLYCOALKALOID METABOLISM 9* (*GAME9*) gene overexpressed or silenced. GAME9 is an APETALA/ethylene response transcription factor located on chromosome 1 in tomato and has been shown to be directly related with the regulation of steroidal alkaloid biosynthesis. Tomato lines overexpressing this transcription factor (*GAME9*-OX) have 2.5-fold increase, whereas silenced lines (*GAME9-*RNAi) have 20-fold reduction in α-tomatine compared to wild type controls^20^. However, there was no significant difference on CPB larval mass (Fig. 6a; F = 0.91, *p* = 0.43) or survival among these lines (Fig. 6b; F = 0.48, *p* = 0.63).

## Discussion

In this study we investigated natural variation in tomato resistance against CPB using two genetic tools developed for tomato, *S. lycopersicum* × *S. pennellii* introgression lines and *S. lycopersicum* × *S. galapagense* RILs. We identified regions in the tomato genome conferring either resistance or enhanced susceptibility to CPB. Overall, our results indicate that, although tomato acylsugar amount and/or composition may have some impact on CPB, they are almost certainly not the most important contributors to CPB resistance in these tomato populations. The tested tomato acylsugar breeding lines with high acylsugar content (FA2/FA7/AS, FA2/AS and FA7/AS, Fig. 5) have a negative effect on larval survival, but not on larval mass or adult beetle emergence. These varying effects would limit the scope of acylsugars in CPB control. Similarly, another beetle from the same family (Chrysomelidae), *Lema daturaphila*, is not affected by the acylsugars of *Datura wrightii*^38^.

Some of the *S. lycopersicum* × *S. pennellii* introgression lines with significant impacts on CPB have previously-identified changes in acylsugar production. For instance, the CPB-resistant introgression line 1–4 has been shown to have an altered acylsucrose profile due to the activity of a specific acyltransferase^15,18^. However, the acylsugar levels in all of the tested introgression lines (Fig. 2) are less than 1% of those found in *S. pennellii* and are lower than those in the tested acylsugar breeding lines (Fig. 5), including that of the low acylsugar control FA5/AS.

GAME9, a transcription factor that is essential for glycoalkaloid biosynthesis in tomato and potato^20^, was localized in the same region as *S. pennellii* introgression 1-1, which is more susceptible to CPB. Although *GAME9* overexpressing plants have increased tomatine levels and silenced plants lack this metabolite, we found no significant effect on CPB larval mass or survival (Fig. 6). The effect of glycoalkaloids on CPB has been studied before with very variable results, ranging from antifeedant and negative effects on development^39,40^, to no effect at all^41,42^. The effect of glycoalkaloids also may be modified by other metabolites in the plant^43^. *GAME9* overexpression and silencing affects other tomato metabolites, including cholesterol and β-amyrin^20^. Thus, the effect on CPB may be due to the interaction of several factors, rather than solely the high or very low levels of tomatine in the *GAME9* mutant lines.

Introgression lines 6-2 and 6-2-2 were the most susceptible to CPB. Moreover, during the experiments these lines showed a weak, stressed phenotype in growth chambers, as is also described by the Tomato Genetic Resource Center (http://tgrc.ucdavis.edu/). A recent study on the expression quantitative trait loci (eQTL, defined as a chromosomal region that drives variation in gene expression patterns) showed that tomato chromosomes 4, 6 and 8 have an abundance of trans eQTLs^44^. Thus, regions on these chromosomes may control the expression of several transcripts. ILs 6-2 and 6-2-2 were identified as regions modifying the expression of genes related to defense and IL8-1-3, one of the introgression lines we found as resistant, of genes related to leaf development^44^. This altered gene expression in IL 6-2 and 6-2-2, which increases pathogen defense^45^, may also affect the defense response to insects such as CPB.

Some of the genes located within the three QTL mapping intervals that we identified using *S. lycopersicum* × *S. galapagense* recombinant inbred lines were previously reported to be involved with different types of stress responses (Supplementary Table 5). These defense-related genes can serve as candidates for further investigation of the genetic basis of natural variation in tomato resistance to CPB. Even without the identification of specific causative loci, the chromosome 6 QTL region that influenced CPB resistance in both of our genetic mapping approaches has potential utility for breeding cultivated tomato varieties with enhanced herbivore resistance.

Together, our results show that tomato resistance to CPB is a complex trait, but that there are individual QTL with strong effects. Known loci related to acylsugar production and tomatine accumulation likely do not account for the observed variation in CPB resistance. Additionally, synergy between defense compounds may be more important than any individual compound. Further research will be required to identify the actual genetic basis and molecular mechanisms that underlie the identified CPB resistance QTL.

## Methods

### Plant material and growth conditions

Seeds of *S. lycopersicum* (M82), *S. pennellii* LA0716, and *S. pennellii* introgression lines were obtained from the Tomato Genetics Resource Center (University of California, Davis). Seeds of *S. lycopersicum* cv. UC-204B × *S. galapagense* LA0483 RILs in the F7 generation were provided by Ilan Paran (The Volcani Center, Israel). The acylsugar producing tomato benchmark line CU071026 and backcrossed inbred lines with altered acylsugar chemotypes (FA2/FA7/AS, FA7/AS, FA2/AS, FA8/AS, and FA5/AS) were characterized and described previously^36,37^. Seeds of tomato lines with overexpression or silencing of the *GLYCOALKALID METABOLISM 9* (*GAME9*) gene, as well as seeds from the wild type background (*S. lycopersicum* var. Micro Tom^20^) were provided by Pablo Cardenas and Asaph Aharoni (Weizmann Institute of Science, Israel).

Tomato plants were grown in a growth chamber with a 16:8 h light:dark photoperiod, 180 mmol photons/m^2^/s light intensity at a constant temperature of 23 °C and 60% humidity. *Solanum lycopersicum* × *S. galapagense* RILs were grown in a greenhouse with a 16:8 h light:dark photoperiod and a temperature range from 28 °C day to 20 °C night.

### Colorado potato beetle growth conditions and bioassays

Ten clutches of CPB eggs were obtained from Jennifer Thaler (Cornell University), from a colony that was originally collected from potato in Tompkins County, New York and which had been maintained on potato in the laboratory for one year (about 10 generations). These eggs were used to establish a colony that was maintained on potato (*Solanum tuberosum* var. Russet) plants in a growth chamber with a 16:8 h light:dark photoperiod and a constant temperature of 23 °C.

Herbivory assays involving parental lines, *S. lycopersicum* cv. M82 and *S. pennellii*, were conducted using young plants with eight fully-expanded leaves. Eight CPB larvae, newly hatched and randomly selected from three egg clutches that had matured at the same time, were placed in each plant (M82 n = 7; *S. pennellii* n = 9) and caged in microperforated polypropylene bags (27.9 cm × 50.8 cm; PJP Marketplace). Five and ten days after infestation, larvae were counted and weighed. Adult emergence rate after pupation also was recorded. Herbivory assays involving *S. pennellii* introgression lines were conducted with plants at the reproductive stage (first floral bud visible). Assays were performed as described above. Ten days after infestation, surviving larvae were counted and weighed. Two or three replicate plants were used for each introgression line.

For herbivory assays using *S. lycopersicum* × *S. galapagense* RILs, 119 individual RILs were sampled. A leaflet of fully expanded leaves from fruiting-stage plants were placed on petri dishes with seven newly hatched CPB larvae. After 5 days of feeding, larval survival and mass were recorded. Damage in the leaflets was also recorded on a categorical scale from 0 to 5, where 0 signified no visible damage and 5 signified at least 50% of the leaflet damaged. The experiment was repeated two additional times with four of the most sensitive RILs and two of the most resistant RILs.

Herbivory assays involving tomato lines with altered acylsugar content were performed as described above for *S. pennellii* introgression lines. Line CU071026 is a benchmark acylsugar breeding line, which contains five *S. pennellii* introgressions, and produces acylsugars at levels at approximately 15% of *S. pennellii* levels^46^. The other acylsugar lines were bred from CU071026. Each of those lines has all five of the *S. pennellii* introgressions of CU071026 and 1 or 2 additional *S. pennellii* introgressions that carry acylsugar QTL^36^. The resulting lines differ from CU071026 for acylsugar accumulation and/or composition^37^. Relative to CU071026, the acylsugar content of FA2/FA7/AS is 171%, FA7/AS and FA2/AS is 121%, FA8/AS is 80%, and FA5/AS is only 16%. Due to its extremely low acylsugar content, line FA5/AS was used as a low/negative acylsugar control. Ten plants were used for each line, with the exception of FA2/FA7/AS (n = 6) and FA2/AS (n = 8), for which the germination rate was lower. Ten days after infestation, surviving larvae were counted and weighed. Adult emergence rate, adult weight, and days to reach the adult stage also were recorded.

Herbivory assays with *GAME9* overexpressing and RNAi-silenced plants were performed as described for *S. pennellii* introgression lines. Four to five plants of each line (wild type, *GAME9*-overexpressing and *GAME9*-RNAi) were used. Larval weight and survival were recorded five days after the start of infestation.

### Quantitative trait locus mapping

SNP marker data for 113 *S. lycopersicum* × *S. galapagense* RILs were generated from fruit pericarp RNA sequencing data. Strand-specific RNA sequencing was performed as described previously^47^. Reads of each line were aligned to the tomato genome (v. 2.50) using STAR (v. 020201)^48^ with “2-pass Mapping” method. GATK (v. 3.4–46)^49^ pipeline was used to call SNPs for each sample, using “HaplotypeCaller” method with parameters: “-GQB 5 -GQB 20 -GQB 60 -GQB 99 -stand_call_conf 20.0 -stand_emit_conf 20.0”. Only sites presenting homozygous differences between the two parental lines were kept for further analysis. A total of 17,567 SNPs were identified and 2,216 genomic bins were calculated and used as genetic markers for QTL analysis.

QTL analysis was performed with WinQTL Cartographer version 2.5^50^. The experimental LOD threshold was determined by permutation tests with 1000 permutations at 0.05 confidence level. Analysis was done by composite interval mapping with the following settings: map function = Kosambi, CIM program module = Model 6: standard model, walking speed, 1 CM, control marker numbers = 5, window size = 10 CM, regression method = backward regression method. Chromosomal confidence intervals to identifying candidate genes for mapped QTL were defined as two LOD unites from the peaks of the respective QTL.

### Quantitative real-time PCR

GAME9 gene expression analysis of the overexpressing and RNAi silenced plants was performed with three biological replicates. RNA isolation was performed with the SV Total RNA Isolation System Kit (Promega). cDNA was obtained using Ultrapure Smart MMVL reverse transcriptase (Clontech). Gene-specific oligonucleotide primers reported in Cárdenas *et al*. (2015) were used for amplifying GAME9 and the endogenous TIP41 genes.

### Statistical analysis

Statistical comparisons were conducted using R software^51^. Correlation tests were conducted using the Kendall’s rank correlation tau (τ) for non-parametric data. Dunnett’s test were conducted using the Multcomp package^52^. Graphs were made in Excel 2007.

### Data availability

The datasets generated during and/or analysed during the current study are available as supplementary materials. Supplementary Dataset 1 is an Excel file that contains data that were used as input for Windows QTL Cartographer^50^ to generate Fig. 4e. Raw data for all other figures are in the Excel file Supplementary Dataset 2.

## Electronic supplementary material

Supplementary Tables 1-5

Dataset 1

Dataset 2
