# Supplementary material for: Genetic mapping identifies loci that influence tomato resistance against Colorado potato beetles
Source: Sci Rep. 2018 May 9;8:7429. doi: 10.1038/s41598-018-24998-5 (PMC5943291; doi:10.1038/s41598-018-24998-5)
Supplement: Supplementary file 1 — Supplementary Tables 1-5 [file 41598_2018_24998_MOESM1_ESM.doc]

**Genetic mapping identifies loci that influence tomato resistance against Colorado potato beetles**

Erandi Vargas-Ortiz^1^, Itay Gonda^1^, John R. Smeda^2^, Martha A. Mutschler^2^, James J. Giovannoni^1,3^ , Georg Jander^1^*

^1^Boyce Thompson Institute, Ithaca, New York 14853, USA; ^2^Department of Plant Breeding and Genetics, Cornell University, Ithaca, NY 14853, USA; ^3^USDA Robert W. Holley Center for Agriculture and Health, Ithaca, New York, 14853, USA.

*To whom correspondence should be addressed:

Georg Jander

Boyce Thompson Institute

533 Tower Road

Ithaca, NY 14853

USA

Phone: 607-254-1365

Email: gj32@cornell.edu

Supplementary Table 1. Markers defining the positions of the identified QTLs (genome version: Tomato SL2.5 ITAG 2.4)

| **QTL** | **SNP^a^** | **Sequence** |
| --- | --- | --- |
| **Leaf damage QTL Ch. 6** | | |
| Flanking marker | SL2.50ch06_45624963 | >SL2.50ch06:45624938..45624987 CAGCCATTGTTAATCGATTTTAGAGCTCTTCTTCTTCTCAAAAAGTGGTC |
| Peak | SL2.50ch06_46776087 | >SL2.50ch06:46776062..46776111 AAGAAAGCATTTTCTTCGAATAAGACAACATGACAAACATAGGAAAATTC |
| Flanking marker | SL2.50ch06_47490321 | >SL2.50ch06:47490296..47490345 CCATGTCTGTTAGATCAGGAGAAAGACCACCATGCATGCAAAGTATTTTA |
| **Leaf damage QTL Ch. 8** | | |
| Flanking marker | SL2.50ch08_61690905 | >SL2.50ch08:61690880..61690929 TGTGATTTAGTGATTTTGGATTGGATAAAATGGAGGAAAAATATGAGCTT |
| Peak | SL2.50ch08_62074703 | >SL2.50ch08:62074678..62074727 GCATCGTGGAGTCAAACGATTTACAAAGGAGCTAAGTGAATGGTCAAAAT |
| Flanking marker | SL2.50ch08_62353298 | >SL2.50ch08:62353273..62353322 TTTGATGGAAATGCAAGATTTTGTCGTAGGCAAGGCAGTTCTGTTTAATG |
| **Larvae mass QTL Ch.6** | | |
| Flanking marker | SL2.50ch06_43514856 | > SL2.50ch06:43514831..43514880 AGTTTTTGAGTCATCCTGTTCTTGGTGACCTTCTAACTGAAGAGGACCAA |
| Peak | SL2.50ch06_44041467 | >SL2.50ch06:44041442..44041491 GTTATGATCGCAAGCAGTCAGGTTACGGTGGACAGACAAAACCTGTCTTT |
| Flanking marker | SL2.50ch06_44760870 | >SL2.50ch06:44760845..44760894 TATTCTCTTGTAACAACATTTTCTTTAAGGGAAAAAAGCTGTTAAGTATT |

^a^ Markers that flank the 2-LOD confidence interval.

Supplementary Table 2 Genes in the leaf damage QTL on Chromosome 6

| **Gene ID** | **Annotation** |
| --- | --- |
| Solyc06g073980.2 | Unknown Protein |
| Solyc06g073990.1 | Unknown Protein |
| Solyc06g074000.1 | Aspartic proteinase nepenthesin-2 |
| Solyc06g074010.2 | Transcription factor (E2F) |
| Solyc06g074020.2 | Os04g0415100 protein (Fragment) |
| Solyc06g074030.1 | CCR4-NOT transcription complex subunit 7 |
| Solyc06g074040.1 | NHL1 |
| Solyc06g074050.1 | Hairpin-induced 1 |
| Solyc06g074060.1 | Unknown Protein |
| Solyc06g074070.2 | Receptor like kinase |
| Solyc06g074080.2 | Histone deacetylase |
| Solyc06g074090.2 | Sterol reductase |
| Solyc06g074100.1 | Aluminum-activated malate transporter |
| Solyc06g074110.2 | Uncharacterized basic helix-loop-helix protein At1g64625 |
| Solyc06g074120.2 | BEL1-like homeodomain protein 1 |
| Solyc06g074130.2 | Growth inhibition and differentiation-related protein 88 |
| Solyc06g074140.1 | U-box domain-containing protein 24 |
| Solyc06g074150.2 | LRR receptor-like serine/threonine-protein kinase |
| Solyc06g074160.1 | B3 domain-containing protein Os03g0212300 |
| Solyc06g074170.2 | NAC domain protein IPR003441 |
| Solyc06g074180.1 | Cytochrome P450 |
| Solyc06g074190.2 | Os04g0625000 protein (Fragment) |
| Solyc06g074200.2 | Sex-linked protein 9 (Fragment) |
| Solyc06g074210.2 | MRNA decapping enzyme |
| Solyc06g074220.2 | OTU domain containing protein |
| Solyc06g074230.2 | membrane protein 1 |
| Solyc06g074240.1 | Chromoplast-specific lycopene beta-cyclase |
| Solyc06g074250.2 | Vesicle-associated membrane protein 7C |
| Solyc06g074260.2 | Genomic DNA chromosome 5 P1 clone MWD9 |
| Solyc06g074270.1 | Genomic DNA chromosome 5 P1 clone MWD9 |
| Solyc06g074280.2 | Ankyrin repeat protein |
| Solyc06g074290.1 | Unknown Protein |
| Solyc06g074300.2 | Ribosomal protein |
| Solyc06g074310.2 | Transmembrane 9 superfamily protein member 1 |
| Solyc06g074320.2 | BZIP transcription factor |
| Solyc06g074330.2 | Hydrolase alpha/beta fold family protein |
| Solyc06g074340.2 | Hydrolase alpha/beta fold family protein |
| Solyc06g074350.2 | self-pruning |
| Solyc06g074360.2 | Zinc finger protein |
| Solyc06g074370.1 | Alpha-2 3-sialyltransferase |
| Solyc06g074380.2 | Methyltransferase domain family |
| Solyc06g074390.2 | Fatty acyl coA reductase |
| Solyc06g074400.2 | Pentatricopeptide repeat-containing protein At2g32230, mitochondrial |
| Solyc06g074410.2 | Fatty acyl coA reductase |
| Solyc06g074420.1 | Cytochrome P450 |
| Solyc06g074430.2 | 60s acidic ribosomal protein-like protein |
| Solyc06g074440.2 | Unknown Protein |
| Solyc06g074450.1 | FRIGIDA |
| Solyc06g074460.2 | Peroxisomal membrane protein PEX16 |
| Solyc06g074470.2 | Unknown Protein |
| Solyc06g074480.2 | Protein kinase |
| Solyc06g074490.1 | Unknown Protein |
| Solyc06g074500.1 | AT5G28150-like protein |
| Solyc06g074510.2 | 3-bisphosphoglycerate-dependent phosphoglycerate mutase 2 |
| Solyc06g074520.1 | Unknown Protein |
| Solyc06g074530.1 | Prephenate dehydratase |
| Solyc06g074540.2 | Phytosulfokines 5 |
| Solyc06g074550.2 | Unknown Protein |
| Solyc06g074560.1 | Unknown Protein |
| Solyc06g074570.1 | Unknown Protein |
| Solyc06g074580.1 | Unknown Protein |
| Solyc06g074590.1 | Ycf2 |
| Solyc06g074600.1 | DNA-directed RNA polymerase subunit beta&apos&apos |
| Solyc06g074610.1 | Unknown Protein |
| Solyc06g074620.2 | Os02g0448600 protein (Fragment) |
| Solyc06g074630.2 | Cellulose synthase-like C6 glycosyltransferase family 2 |
| Solyc06g074640.1 | Chromodomain helicase-DNA-binding protein 3 |
| Solyc06g074650.2 | AP-2 complex subunit alpha |
| Solyc06g074660.1 | Unknown Protein |
| Solyc06g074670.2 | Bifunctional polymyxin resistance arnA protein |
| Solyc06g074680.2 | Phospholipid diacylglycerol acyltransferase |
| Solyc06g074690.2 | Genomic DNA chromosome 5 P1 clone MRN17 |
| Solyc06g074700.2 | Serine/threonine protein kinase |
| Solyc06g074710.1 | Hydroxycinnamoyl CoA shikimate/quinate hydroxycinnamoyltransferase-like protein |
| Solyc06g074720.2 | MKI67 FHA domain-interacting nucleolar phosphoprotein-like |
| Solyc06g074730.2 | ARGONAUTE 1 |
| Solyc06g074740.2 | Vacuolar protein sorting-associated protein 41 homolog |
| Solyc06g074750.1 | Histone H2B |
| Solyc06g074760.1 | Zinc finger, RING-type |
| Solyc06g074770.2 | Ubiquitin conjugation factor E4 |
| Solyc06g074780.1 | Histone H2B |
| Solyc06g074790.1 | Histone H2B |
| Solyc06g074800.1 | Cys2/His2 zinc-finger transcription factor |
| Solyc06g074810.2 | Protein SMG8 |
| Solyc06g074820.2 | Aquaporin-like protein |
| Solyc06g074830.1 | Unknown Protein |
| Solyc06g074840.2 | Charged multivesicular body protein 3 |
| Solyc06g074850.2 | Serine carboxypeptidase |
| Solyc06g074860.1 | Double-strand-break repair protein rad21 |
| Solyc06g074870.1 | Sister chromatid cohesion 1 protein 1 |
| Solyc06g074880.1 | Unknown Protein |
| Solyc06g074890.1 | Trigger factor |
| Solyc06g074900.1 | Trigger factor |
| Solyc06g074910.2 | MYB transcription factor |
| Solyc06g074920.1 | MYB transcription factor |
| Solyc06g074930.1 | Metal ion binding protein |
| Solyc06g074940.2 | ATP-binding cassette protein |
| Solyc06g074950.1 | Auxin-responsive family protein |
| Solyc06g074960.2 | ABC transporter G family member 3 |
| Solyc06g074970.1 | ABC transporter G family member 3 |
| Solyc06g074980.2 | 26S protease regulatory subunit 6B homolog |
| Solyc06g074990.1 | Nitrate transporter |
| Solyc06g075000.2 | MaoC-like dehydratase |
| Solyc06g075010.2 | Chaperonin |
| Solyc06g075020.2 | ABC transporter G family member 28 |
| Solyc06g075030.1 | Receptor-like kinase |
| Solyc06g075040.1 | Unknown Protein |
| Solyc06g075050.1 | Carbonic anhydrase |
| Solyc06g075060.1 | Carbonic anhydrase |
| Solyc06g075070.2 | Carbonic anhydrase |
| Solyc06g075080.2 | NADH pyrophosphatase |
| Solyc06g075090.2 | Lysine decarboxylase-like protein |
| Solyc06g075100.2 | Glycosyl transferase family 2 protein |
| Solyc06g075110.2 | Lysine ketoglutarate reductase trans-splicing related 1 |
| Solyc06g075120.1 | Unknown Protein |
| Solyc06g075130.2 | Kinase-START domain protein |
| Solyc06g075140.2 | GATA transcription factor 1 |
| Solyc06g075150.2 | Auxin response factor 16 |
| Solyc06g075160.2 | Serine/threonine-protein phosphatase 6 regulatory subunit 3 |
| Solyc06g075170.1 | Arabidopsis thaliana genomic DNA chromosome 5 P1 clone MOK16 |
| Solyc06g075180.1 | Ribosomal protein L12 |
| Solyc06g075190.1 | Unknown Protein |
| Solyc06g075200.1 | Unknown Protein |
| Solyc06g075210.2 | Protein disulfide isomerase L-2 |
| Solyc06g075220.1 | ,Fasciclin-like arabinogalactan protein 5 |
| Solyc06g075240.1 | Ulp1 protease family C-terminal catalytic domain |
| Solyc06g075250.2 | C2H2L domain class transcription factor |
| Solyc06g075260.2 | Vicilin (Fragment) |
| Solyc06g075270.2 | Convicilin (Fragment) |
| Solyc06g075280.2 | Vicilin (Fragment) |
| Solyc06g075290.2 | globulin-3 (Fragment) |
| Solyc06g075300.1 | Vicilin (Fragment) |
| Solyc06g075310.2 | Adenylate kinase isoenzyme 6 |
| Solyc06g075320.2 | Vicilin (Fragment) |
| Solyc06g075330.1 | LOB domain protein 1 |
| Solyc06g075340.2 | Carbamoyl-phosphate synthase large chain |
| Solyc06g075350.1 | tRNA-splicing endonuclease |
| Solyc06g075360.2 | Senescence-associated protein |
| Solyc06g075370.2 | Dof zinc finger protein 9 |
| Solyc06g075380.1 | Unknown Protein |
| Solyc06g075390.2 | Exosome complex exonuclease RRP43 |
| Solyc06g075400.2 | V-type proton ATPase subunit a |
| Solyc06g075410.2 | Unknown Protein |
| Solyc06g075420.1 | BAC clone F15L12 |
| Solyc06g075430.1 | Unknown Protein |
| Solyc06g075440.1 | Peptide transporter |
| Solyc06g075450.1 | Peptide transporter 1 |
| Solyc06g075460.2 | TGF-beta receptor type I/II extracellular region |
| Solyc06g075470.1 | Nucleoside diphosphate kinase |
| Solyc06g075480.2 | Nitrate transporter |
| Solyc06g075490.1 | Nitrate transporter |
| Solyc06g075500.2 | Nitrate transporter |
| Solyc06g075510.2 | AP2-like ethylene-responsive transcription factor At1g16060 |
| Solyc06g075520.2 | Dehydroascorbate reductase 1 |
| Solyc06g075530.1 | Unknown Protein |
| Solyc06g075540.2 | Phosphatidyl synthase |
| Solyc06g075550.2 | Serine/threonine kinase |
| Solyc06g075560.1 | Glycosyltransferase-like protein |
| Solyc06g075570.1 | espiratory burst oxidase protein A |
| Solyc06g075580.2 | Kinesin-5 |
| Solyc06g075590.2 | NOT transcription complex subunit 2 |
| Solyc06g075600.2 | Ankyrin repeat family protein |
| Solyc06g075610.1 | Exocyst complex component 7 |
| Solyc06g075620.2 | 60S ribosomal protein L35 |
| Solyc06g075630.2 | Dirigent-like protein |
| Solyc06g075640.1 | Male sterility 5 family protein |
| Solyc06g075650.2 | Aquapori |
| Solyc06g075660.2 | MYB transcription factor |
| Solyc06g075670.1 | MYB transcription factor |
| Solyc06g075680.1 | RNase H family protein |
| Solyc06g075690.2 | Auxin-regulated protein |
| Solyc06g075700.2 | Unknown Protein |
| Solyc06g075710.1 | Unknown Protein |
| Solyc06g075720.1 | Unknown Protein |
| Solyc06g075730.1 | Unknown Protein |
| Solyc06g075740.1 | Unknown Protein |
| Solyc06g075750.1 | Unknown Protein |
| Solyc06g075760.1 | Unknown Protein |
| Solyc06g075770.1 | Unknown Protein |
| Solyc06g075780.1 | Unknown Protein |
| Solyc06g075790.2 | Genomic DNA chromosome 5 P1 clone MMN10 |
| Solyc06g075800.1 | Histone H2B |
| Solyc06g075810.2 | NADH dehydrogenase |
| Solyc06g075820.1 | Protein yippee-like |
| Solyc06g075830.1 | Histone H4 |
| Solyc06g075840.1 | Unknown Protein |
| Solyc06g075850.1 | Histone H4 |
| Solyc06g075860.1 | Protein yippee-like |
| Solyc06g075870.1 | Unknown Protein |
| Solyc06g075880.1 | Unknown Protein |
| Solyc06g075890.1 | Unknown Protein |
| Solyc06g075900.1 | Erythrocyte membrane-associated giant protein antigen 332 |
| Solyc06g075910.1 | Unknown Protein |
| Solyc06g075920.1 | Unknown Protein |
| Solyc06g075930.1 | Histone H4 |
| Solyc06g075940.1 | Unknown Protein |
| Solyc06g075950.1 | Protein yippee-like |
| Solyc06g075960.1 | Histone H4 |
| Solyc06g075970.2 | Unknown Protein |
| Solyc06g075980.2 | CRAL/TRIO domain containing protein |
| Solyc06g075990.2 | Glyoxalase/bleomycin resistance protein/dioxygenase |
| Solyc06g076000.1 | WUSCHEL-related homeobox-containing protein 4 |
| Solyc06g076010.2 | Unknown Protein |
| Solyc06g076020.2 | heat shock protein |
| Solyc06g076030.2 | Dof zinc finger protein |
| Solyc06g076040.2 | U-box domain-containing protein |
| Solyc06g076050.2 | Ankyrin repeat domain-containing protein 28 |
| Solyc06g076060.1 | Unknown Protein |
| Solyc06g076070.1 | Unknown Protein |
| Solyc06g076080.1 | Unknown Protein |
| Solyc06g076090.2 | Actin |
| Solyc06g076100.2 | Protein phosphatase 2C containing protein |
| Solyc06g076110.1 | Fasciclin-like arabinogalactan protein 9 |
| Solyc06g076120.2 | Fasciclin-like arabinogalactan protein 9 |
| Solyc06g076130.2 | Unknown Protein |
| Solyc06g076140.2 | Metallothionein-like protein |
| Solyc06g076150.2 | Eukaryotic translation initiation factor 3 subunit 8-like protein |
| Solyc06g076160.2 | Cytochrome P450, |
| Solyc06g076170.2 | Glucan endo-1 3-beta-glucosidase |
| Solyc06g076180.1 | Unknown Protein |
| Solyc06g076190.1 | Ulp1 protease family C-terminal catalytic domain containing protein |
| Solyc06g076200.1 | X1 (Fragment) |
| Solyc06g076210.1 | X1 (Fragment) |
| Solyc06g076220.2 | Expansin-1 |
| Solyc06g076230.1 | Unknown Protein |
| Solyc06g076240.1 | Unknown Protein |
| Solyc06g076250.2 | Genomic DNA chromosome 5 BAC clone F2O15 |
| Solyc06g076260.2 | C20orf24 homolog |
| Solyc06g076270.2 | Telomere repeat-binding protein 5 |
| Solyc06g076280.1 | GRAS family transcription factor |
| Solyc06g076290.1 | GRAS family transcription factor |
| Solyc06g076300.2 | Pyrimidine 5&apos-nucleotidase |
| Solyc06g076310.1 | Unknown Protein |
| Solyc06g076320.1 | DVL13 |
| Solyc06g076330.2 | Laccase |
| Solyc06g076340.2 | mRNA binding protein Pumilio 2 |
| Solyc06g076350.2 | Transcription factor (Fragment) |

Supplementary Table 3. Genes in the leaf damage QTL on chromosome 8

| **Gene ID** | **Annotation** |
| --- | --- |
| Solyc08g077790.2 | Peptidyl-prolyl cis-trans isomerase-like 3 |
| Solyc08g077800.2 | DNA repair endonuclease XPF |
| Solyc08g077810.1 | Cationic amino acid transporter |
| Solyc08g077820.2 | Cationic amino acid transporter |
| Solyc08g077830.1 | Deoxyhypusine hydroxylase |
| Solyc08g077840.2 | Cupin 2 conserved barrel domain protein |
| Solyc08g077850.1 | tRNA/rRNA methyltransferase SpoU family protein |
| Solyc08g077860.2 | Subtilisin-like serine protease |
| Solyc08g077870.2 | Unknown Protein |
| Solyc08g077880.2 | Light harvesting-like protein 3 |
| Solyc08g077890.2 | ATP-dependent Clp protease proteolytic subunit |
| Solyc08g077900.2 | Expansin-like protein |
| Solyc08g077910.2 | Expansin-like protein |
| Solyc08g077920.2 | Isocitrate dehydrogenase |
| Solyc08g077930.2 | Isocitrate dehydrogenase |
| Solyc08g077940.1 | Histone-lysine N-methyltransferase-like protein |
| Solyc08g077950.1 | Unknown Protein |
| Solyc08g077960.2 | GATA transcription factor 29 |
| Solyc08g077970.2 | Unknown Protein |
| Solyc08g077980.2 | Bax inhibitor |
| Solyc08g077990.2 | Kinase like protein |
| Solyc08g078000.2 | Vesicle-associated membrane family protein |
| Solyc08g078010.2 | 50S ribosomal protein L19 |
| Solyc08g078020.1 | Methionine rich arabinogalactan |
| Solyc08g078030.2 | Hydroxycinnamoyl CoA shikimate/quinate hydroxycinnamoyltransferase |
| Solyc08g078040.2 | Monooxygenase FAD-binding |
| Solyc08g078050.1 | CTF2A |
| Solyc08g078060.2 | Unknown Protein |
| Solyc08g078070.2 | Ras-related protein Rab-1A |
| Solyc08g078080.2 | Pentatricopeptide repeat-containing protein |
| Solyc08g078090.1 | Lipase (Fragment) |
| Solyc08g078100.1 | Amino acid permease-like protein |
| Solyc08g078110.1 | Unknown Protein |
| Solyc08g078120.1 | Chromodomain helicase DNA binding protein 3 (Fragment) |
| Solyc08g078130.2 | Fatty acid oxidation complex subunit alpha |
| Solyc08g078140.2 | Ubiquitin carboxyl-terminal hydrolase isozyme L3 |
| Solyc08g078150.1 | Pentatricopeptide repeat-containing protein |
| Solyc08g078160.2 | Oleosin |
| Solyc08g078170.1 | Ethylene responsive transcription factor 1a |
| Solyc08g078180.1 | Ethylene responsive transcription factor 1a |
| Solyc08g078190.1 | Ethylene responsive transcription factor 1a |
| Solyc08g078200.1 | H-ATPase |
| Solyc08g078210.2 | Hydrolase NUDIX family protein |
| Solyc08g078220.2 | PPPDE peptidase domain-containing protein 1 |
| Solyc08g078230.2 | Zinc ion binding protein |
| Solyc08g078240.2 | Unknown Protein |
| Solyc08g078250.2 | Palmitoyl protein thioesterase family protein |
| Solyc08g078260.1 | Palmitoyl protein thioesterase family protein |
| Solyc08g078270.2 | Dual specificity protein phosphatase family protein |
| Solyc08g078280.1 | Pentatricopeptide repeat-containing protein |
| Solyc08g078290.1 | Glycoprotein homolog |
| Solyc08g078300.2 | Homeobox-leucine zipper protein |
| Solyc08g078310.2 | Unknown Protein |
| Solyc08g078320.2 | E3 ubiquitin-protein ligase HOS1 |
| Solyc08g078330.2 | Oxidoreductase 2OG-Fe(II) oxygenase family |
| Solyc08g078340.2 | Myb family transcription factor |
| Solyc08g078350.1 | Unknown Protein |
| Solyc08g078360.1 | Unknown Protein |
| Solyc08g078370.2 | Kinase pfkB family protein |
| Solyc08g078380.2 | CigA protein |
| Solyc08g078390.2 | Acyl-coenzyme A oxidase |
| Solyc08g078400.2 | Acyl-CoA oxidase |
| Solyc08g078410.1 | Ethylene-responsive transcription factor 1 |
| Solyc08g078420.1 | Ethylene-responsive transcription factor 1 |
| Solyc08g078430.2 | Pre-mRNA processing ribonucleoprotein binding region-containing protein |
| Solyc08g078440.2 | Genomic DNA chromosome 5 P1 clone MQL5 |
| Solyc08g078450.2 | Exostosin family protein |
| Solyc08g078460.2 | Inositol 2-dehydrogenase like protein |
| Solyc08g078470.2 | FHA domain containing protein expressed |
| Solyc08g078480.2 | Unknown Protein |
| Solyc08g078490.2 | Formyltetrahydrofolate deformylase |
| Solyc08g078500.2 | Os02g0658033 protein (Fragment) |
| Solyc08g078510.2 | GRAM-containing/ABA-responsive protein (Fragment) |
| Solyc08g078520.2 | Os03g0859900 protein (Fragment) |
| Solyc08g078530.2 | Agenet domain containing protein expressed |
| Solyc08g078540.2 | Os03g0169000 protein |

Supplementary Table 4. Genes in the larval mass QTL on Chromosome 6

| **Gene ID** | **Annotation** |
| --- | --- |
| Solyc06g071000.2 | N-succinylglutamate 5-semialdehyde dehydrogenase |
| Solyc06g071010.2 | Unknown Protein |
| Solyc06g071020.2 | Pectate lyase |
| Solyc06g071030.2 | Isochorismate synthase |
| Solyc06g071040.2 | YLP motif containing 1 |
| Solyc06g071050.2 | Spfh domain / band 7 family protein |
| Solyc06g071060.1 | Short-chain dehydrogenase/reductase family protein |
| Solyc06g071070.1 | Short-chain dehydrogenase/reductase family protein |
| Solyc06g071080.2 | Proton-dependent oligopeptide transport family protein |
| Solyc06g071090.1 | Low affinity nitrate transporter |
| Solyc06g071100.2 | H-ATPase |
| Solyc06g071110.2 | Chaperone protein dnaJ 3 |
| Solyc06g071120.2 | Ubiquitin carboxyl-terminal hydrolase family protein expressed |
| Solyc06g071130.2 | CHY zinc finger containing protein |
| Solyc06g071140.2 | EARLY FLOWERING 5 |
| Solyc06g071150.2 | Hedgehog-interacting protein 1 (Fragment) |
| Solyc06g071160.2 | Beta-1 3-galactosyltransferase-like protein |
| Solyc06g071170.2 | Unknown Protein |
| Solyc06g071180.2 | Dynein light chain 1 cytoplasmic |
| Solyc06g071190.2 | IFA binding protein |
| Solyc06g071200.2 | Importin 13 |
| Solyc06g071210.2 | Ribosomal protein S6 kinase 2 alpha |
| Solyc06g071220.1 | Unknown Protein |
| Solyc06g071230.2 | MYB transcription factor |
| Solyc06g071240.1 | Glutamine-dependent NAD+ synthetase |
| Solyc06g071250.2 | Unknown Protein |
| Solyc06g071260.2 | AT3g47831/T23J7 |
| Solyc06g071270.2 | CCR4-NOT transcription complex subunit 4 |
| Solyc06g071280.2 | Enhanced disease susceptibility 1 (Fragment) |
| Solyc06g071290.2 | Betaine aldehyde dehydrogenase |
| Solyc06g071300.1 | Genomic DNA chromosome 5 P1 clone MSN9 |
| Solyc06g071310.2 | LIM domain protein |
| Solyc06g071320.2 | Genomic DNA chromosome 5 P1 clone MQB2 |
| Solyc06g071330.2 | Nucleobase ascorbate transporter |
| Solyc06g071340.1 | Unknown Protein |
| Solyc06g071350.2 | Os02g0742100 protein (Fragment) |
| Solyc06g071580.2 | MORC family CW-type zinc finger 3 |
| Solyc06g071590.2 | Carboxyl-terminal proteinase |
| Solyc06g071600.2 | Kinetochore protein Spc25 |
| Solyc06g071610.1 | Retinol dehydrogenase 12 |
| Solyc06g071620.2 | Related to ATP dependent RNA helicase |
| Solyc06g071630.1 | Integrator complex subunit 3 homolog |
| Solyc06g071640.2 | Alliinase (Fragment) |
| Solyc06g071650.1 | AP-1 complex subunit mu |
| Solyc06g071660.1 | LOB domain protein |
| Solyc06g071670.1 | Genomic DNA chromosome 5 TAC clone K15C23 |
| Solyc06g071680.2 | Histone deacetylase |
| Solyc06g071690.2 | Myb transcription factor |
| Solyc06g071700.1 | Primary amine oxidase |
| Solyc06g071710.1 | Unknown Protein |
| Solyc06g071720.1 | 60S ribosomal protein L27A |
| Solyc06g071730.2 | Unknown Protein |
| Solyc06g071740.1 | Unknown Protein |
| Solyc06g071750.2 | Octicosapeptide/Phox/Bem1p domain-containing protein |
| Solyc06g071760.1 | Unknown Protein |
| Solyc06g071770.2 | ZZ type zinc finger domain-containing protein |
| Solyc06g071780.2 | Protein DEHYDRATION-INDUCED 19 homolog 5 |
| Solyc06g071790.2 | Elongation factor Tu |
| Solyc06g071800.2 | Serine/threonine protein kinase |
| Solyc06g071810.1 | Receptor like kinase |
| Solyc06g071820.2 | Speckle-type poz protein |
| Solyc06g071830.2 | BTB/POZ domain-containing protein |
| Solyc06g071840.1 | Pectate lyase |
| Solyc06g071850.2 | TPR repeat |
| Solyc06g071860.2 | Zinc finger CCCH domain-containing protein 67 |
| Solyc06g071870.2 | 60S ribosomal protein L17 |
| Solyc06g071880.2 | 60S ribosomal protein L17 |
| Solyc06g071890.2 | Brain protein 44-like protein |
| Solyc06g071900.2 | Pentatricopeptide repeat-containing protein |
| Solyc06g071910.2 | 3-oxoacyl-reductase |
| Solyc06g071920.2 | Glyceraldehyde-3-phosphate dehydrogenase |
| Solyc06g071930.1 | Unknown Protein |
| Solyc06g071940.1 | Salutaridinol 7-O-acetyltransferase |
| Solyc06g071950.1 | At5g19980 (Fragment) |
| Solyc06g071960.2 | Nucleoside diphosphate kinase |
| Solyc06g071970.1 | Unknown Protein |
| Solyc06g071980.2 | Cell division protease ftsH |
| Solyc06g071990.2 | AAA-type ATPase family protein |
| Solyc06g072000.1 | Katanin p60 ATPase-containing subunit A-like 1 |
| Solyc06g072010.1 | Predicted ATPase |
| Solyc06g072020.1 | Unknown Protein |
| Solyc06g072030.1 | Unknown Protein |
| Solyc06g072040.1 | Nuclear transcription factor Y subunit C-1 |
| Solyc06g072050.2 | CBS domain containing protein |
| Solyc06g072060.1 | Os02g0508100 protein |
| Solyc06g072070.1 | Os02g0508100 protein |
| Solyc06g072080.2 | Sumo activating enzyme 1b |
| Solyc06g072090.1 | ABC transporter G family member 11 |
| Solyc06g072100.1 | ABC transporter G family member 11 |
| Solyc06g072110.2 | Tex2 protein |
| Solyc06g072120.2 | 40S ribosomal protein SA |
| Solyc06g072130.2 | Aquaporin |
| Solyc06g072140.2 | Aquaporin |
| Solyc06g072160.2 | Alcohol dehydrogenase 1 |
| Solyc06g072170.2 | Coiled-coil domain-containing protein 12 |
| Solyc06g072180.1 | Agenet domain-containing protein |
| Solyc06g072190.1 | Unknown Protein |
| Solyc06g072200.1 | Agenet domain-containing protein |
| Solyc06g072210.1 | Kunitz trypsin inhibitor |
| Solyc06g072220.1 | Kunitz trypsin inhibitor |
| Solyc06g072230.1 | Kunitz trypsin inhibitor |
| Solyc06g072240.1 | Histone H4 |
| Solyc06g072250.2 | Methyltransferase WBSCR22 |
| Solyc06g072260.1 | Unknown Protein |
| Solyc06g072270.2 | RING finger protein 24 |
| Solyc06g072280.2 | Small nuclear ribonucleoprotein E |
| Solyc06g072290.2 | Protein Kinase interacting protein |
| Solyc06g072300.2 | ARGONAUTE 1 |
| Solyc06g072310.2 | Homeobox-leucine zipper protein PROTODERMAL FACTOR 2 |
| Solyc06g072320.2 | 8-amino-7-oxononanoate synthase |
| Solyc06g072330.2 | Mitochondrial import inner membrane translocase subunit TIM14 |
| Solyc06g072340.2 | Kinase family protein |
| Solyc06g072350.2 | UPF0497 membrane protein 17 |
| Solyc06g072360.2 | Zinc finger protein |
| Solyc06g072370.2 | GAGA-binding transcriptional activator |
| Solyc06g072380.2 | Dihydroxyacetone/glycerone kinase-like protein |
| Solyc06g072390.2 | Inositol 1 4 5-trisphosphate 5-phosphatase-like protein |
| Solyc06g072400.1 | Inositol 1 4 5-trisphosphate 5-phosphatase-like protein |
| Solyc06g072410.1 | Unknown Protein |
| Solyc06g072420.2 | Acid phosphatase |
| Solyc06g072430.1 | Bcl-2-associated athanogene-like protein |
| Solyc06g072440.2 | Erwinia induced protein 2 |
| Solyc06g072450.1 | Agenet domain-containing protein |
| Solyc06g072460.1 | CHP-rich zinc finger protein-like |
| Solyc06g072470.2 | 50S ribosomal protein L29 |
| Solyc06g072480.1 | Knotted-like homeobox protein |
| Solyc06g072490.2 | 40S ribosomal protein S1 |
| Solyc06g072500.2 | N-acetyltransferase |
| Solyc06g072510.2 | Mitochondrial phosphate carrier protein |
| Solyc06g071370.1 | NHL repeat-containing protein-like |
| Solyc06g071380.2 | Unknown Protein |
| Solyc06g071390.2 | Deleted in split hand/splt foot protein 1 |
| Solyc06g071400.2 | RAG1-activating protein 1 homolog |
| Solyc06g071410.2 | Protein kinase |
| Solyc06g071420.2 | Ring finger protein 12 |
| Solyc06g071430.2 | T17H3.1 protein |
| Solyc06g071440.2 | UPF0497 membrane protein At2g36330 |
| Solyc06g071450.2 | RNA polymerase II transcription factor B subunit 4 |
| Solyc06g071460.2 | Exosome complex exonuclease RRP40 |
| Solyc06g071470.2 | Peroxisomal membrane protein PEX14 |
| Solyc06g071480.2 | Dof zinc finger protein 12 |
| Solyc06g071490.1 | Serine/threonine-protein kinase ATM |
| Solyc06g071500.2 | Boron transporter |
| Solyc06g071510.2 | Isoamyl acetate-hydrolyzing esterase |
| Solyc06g071520.1 | Myb family transcription factor |
| Solyc06g071530.2 | ribosomal protein L44 |
| Solyc06g071540.2 | clone RAFL24-05-D16 |
| Solyc06g071550.2 | Anthranilate phosphoribosyltransferase |
| Solyc06g071560.2 | Serine/threonine protein phosphatase 2A 55 kDa regulatory subunit B beta isoform |
| Solyc06g071570.2 | Solute carrier family 15 member 4 |

Supplementary Table 5, Genes in the three QTL regions that are involved in other stresses.

| **Gene ID** | **Annotation** | **Response to^*^** | **Reference^#^** |
| --- | --- | --- | --- |
| Solyc06g071070.1 | Short-chain dehydrogenase/reductase family protein | FIRE | 2 |
| Solyc06g071540.2 | clone RAFL24-05-D16 | ABA | 5 |
| Solyc06g071560.2 | Serine/threonine protein phosphatase 2A 55 kDa regulatory subunit B beta isoform | ABA | 5 |
| Solyc06g071580.2 | MORC family CW-type zinc finger 3 | ABA | 5 |
| Solyc06g071600.2 | Kinetochore protein Spc25 | ABA | 5 |
| Solyc06g072460.1 | CHP-rich zinc finger protein-like | FIRE | 2 |
| Solyc06g074090.2 | Sterol reductase | TU | 1 |
| Solyc06g074120.2 | BEL1-like homeodomain protein 1 | TU | 1 |
| Solyc06g074130.2 | Growth inhibition and differentiation-related protein 88 | ABA | 5 |
| Solyc06g074190.2 | Os04g0625000 protein | TU | 1 |
| Solyc06g074370.1 | Alpha-2 3-sialyltransferase | TU | 1 |
| Solyc06g074480.2 | Protein kinase | ABA | 5 |
| Solyc06g074530.1 | Prephenate dehydratase | TU | 1 |
| Solyc06g074840.2 | Charged multivesicular body protein 3 | ETI-PTI | 3 |
| Solyc06g074940.2 | ATP-binding cassette protein | ETI | 3 |
| Solyc06g074960.2 | ABC transporter G family member 3 | ETI | 3 |
| Solyc06g074990.1 | Nitrate transporter | ETI | 3 |
| Solyc06g075010.2 | Chaperonin | TU | 1 |
| Solyc06g075090 | Lysine decarboxylase-like protein | CK | 4 |
| Solyc06g075110.2 | Lysine ketoglutarate reductase trans-splicing related 1 | ETI | 3 |
| Solyc06g075170.1 | Arabidopsis thaliana genomic DNA chromosome 5 P1 clone MOK16 | TU | 1 |
| Solyc06g075180.1 | Ribosomal protein L12 | ETI, TU | 3,1 |
| Solyc06g075190.1 | Unknown Protein | ETI-PTI | 3 |
| Solyc06g075310.2 | Adenylate kinase isoenzyme 6 | ETI-PTI | 3 |
| Solyc06g075330.1 | LOB domain protein 1 | TU | 1 |
| Solyc06g075350.1 | tRNA-splicing endonuclease | ABA | 5 |
| Solyc06g075360.2 | Senescence-associated protein | TU | 1 |
| Solyc06g075370.2 | Dof zinc finger protein 9 | ETI | 3 |
| Solyc06g075400.2 | V-type proton ATPase subunit a | ETI | 3 |
| Solyc06g075410 | Unknown protein | CK | 4 |
| Solyc06g075530.1 | Unknown Protein | ETI | 3 |
| Solyc06g075550.2 | Serine/threonine kinase | ETI, TU | 3,1 |
| Solyc06g075600.2 | Ankyrin repeat family protein | ABA | 5 |
| Solyc06g075680.1 | RNase H family protein | ABA | 5 |
| Solyc06g075690.2 | Auxin-regulated protein | ETI, ABA | 3, 5 |
| Solyc06g075780.1 | Unknown Protein | ETI | 3 |
| Solyc06g075800.1 | Histone H2B | ETI | 3 |
| Solyc06g076020.2 | heat shock protein | ETI, TU | 1 |
| Solyc06g076050.2 | Ankyrin repeat domain-containing protein 28 | TU | 44 |
| Solyc06g076080.1 | Unknown Protein | ETI | 46 |
| Solyc06g076100.2 | Protein phosphatase 2C containing protein | ETI | 46 |
| Solyc06g076140.2 | Metallothionein-like protein | ABA | 48 |
| Solyc06g076160.2 | Cytochrome P450 | ETI, TU | 44 |
| Solyc06g076260.2 | C20orf24 homolog | ETI-PTI | 46 |
| Solyc06g076300.2 | Pyrimidine 5&apos-nucleotidase | ETI | 46 |
| Solyc06g076320.1 | DVL13 | ETI | 46 |
| Solyc08g077980.2 | Bax inhibitor | ETI | 46 |
| Solyc08g078000.2 | Vesicle-associated membrane family protein | ETI | 46 |
| Solyc08g078040.2 | Monooxygenase FAD-binding | ETI, FIRE | 45, 46 |
| Solyc08g078050.1 | CTF2A | ETI, FIRE | 45, 46 |
| Solyc08g078060.2 | Unknown Protein | ETI | 46 |
| Solyc08g078070.2 | Ras small GTPase, Rab type | TU | 44 |
| Solyc08g078090.1 | Lipase | TU | 44 |
| Solyc08g078170.1 | Ethylene-responsive transcription factor 1A | FIRE | 45 |
| Solyc08g078180.1 | Ethylene-responsive transcription factor 1A | ETI, | 46 |
| Solyc08g078190.1 | Ethylene responsive transcription factor 1a | ETI, ABA | 46, 48 |
| Solyc08g078210.2 | Hydrolase NUDIX family protein | TU | 44 |
| Solyc08g078310.2 | Unknown Protein | ETI | 46 |
| Solyc08g078370.2 | Kinase pfkB family protein | ETI | 46 |
| Solyc08g078390.2 | Acyl-coenzyme A oxidase | ETI | 46 |
| Solyc08g078400.2 | Acyl-CoA oxidase | ETI | 46 |
| Solyc08g078440.2 | Genomic DNA chromosome 5 P1 clone MQL5 | ETI | 46 |
| Solyc08g078480.2 | Unknown Protein | ETI | 46 |

*Some genes located within the three QTL intervals that were identified by genetic mapping with *Solanum lycopersicum* × *Solanum galapagense* recombinant inbred lines (Figure 3a) previously were reported to be differentially regulated in response to plant stress. Fourteen genes from the leaf damage QTL on chromosome 6 and three on chromosome 8 were differentially expressed after *T. urticae* infestation (marked as TU)[^1^](#_ENREF_1). Three genes from the damage QTL on chromosome 8 and two genes from larva mass QTL on chromosome 6 were identified as FIRE (Flagellin-Induced Repressed by Effectors)[^2^](#_ENREF_2) genes related to plant immunity, (Supplementary Table 5, marked as FIRE). Similarly, eighteen genes within the leaf damage QTL on chromosome 6 were found to be induced specifically by the Effector Triggered Immunity (ETI) as well as thirteen genes within the leaf damage QTL on chromosome 8 (marked as ETI in Supplementary Table 5). Four more genes in the leaf damage QTL chromosome 6 were induced by both ETI and Pattern Triggered Immunity (PTI)[^3^](#_ENREF_3), marked as ETI-PTI. Two genes within the leaf damage QTL on chromosome 6 are repressed by cytokinins (marked as CK) in leaves after treatment with benzyladenine[^4^](#_ENREF_4). Treatment in the leaves with exogenous abscisic acid[^5^](#_ENREF_5) modified the expression of twelve genes among the three found QTL (marked as ABA).

**^#^**References for Supplementary Table 5

1. Martel, C. *et al.* Tomato whole genome transcriptional response to *Tetranychus urticae* identifies divergence of spider mite-induced responses between tomato and arabidopsis. *Molecular Plant-Microbe Interactions* **28**, 343-361, doi:10.1094/mpmi-09-14-0291-fi (2015).

2. Rosli, H. G. *et al.* Transcriptomics-based screen for genes induced by flagellin and repressed by pathogen effectors identifies a cell wall-associated kinase involved in plant immunity. *Genome Biol* **14**, doi:10.1186/gb-2013-14-12-r139 (2013).

3. Pombo, M. A. *et al.* Transcriptomic analysis reveals tomato genes whose expression is induced specifically during effector-triggered immunity and identifies the Epk1 protein kinase which is required for the host response to three bacterial effector proteins. *Genome Biology* **15**, 492, doi:10.1186/s13059-014-0492-1 (2014).

4. Shi, X. *et al.* Transcriptome analysis of cytokinin response in tomato leaves. *PLoS ONE* **8**, e55090, doi:10.1371/journal.pone.0055090 (2013).

5. Wang, Y. *et al.* Comparative transcriptome analysis of tomato (*Solanum lycopersicum*) in response to exogenous abscisic acid. *BMC Genomics* **14**, 841, doi:10.1186/1471-2164-14-841 (2013).
